# A high level interface to SCOP and ASTRAL implemented in Python

**DOI:** 10.1186/1471-2105-7-10

**Published:** 2006-01-10

**Authors:** James A Casbon, Gavin E Crooks, Mansoor AS Saqi

**Affiliations:** 1 Bioinformatics, Institute of Cell and Molecular Science, School of Medicine and Dentistry, Queen Mary, University of London, Charterhouse Square, London EC1 6BQ, UK; 2 Department of Plant and Microbial Biology, University of California, Berkeley, USA

## Abstract

**Background:**

Benchmarking algorithms in structural bioinformatics often involves the construction of datasets of proteins with given sequence and structural properties. The SCOP database is a manually curated structural classification which groups together proteins on the basis of structural similarity. The ASTRAL compendium provides non redundant subsets of SCOP domains on the basis of sequence similarity such that no two domains in a given subset share more than a defined degree of sequence similarity. Taken together these two resources provide a 'ground truth' for assessing structural bioinformatics algorithms. We present a small and easy to use API written in python to enable construction of datasets from these resources.

**Results:**

We have designed a set of python modules to provide an abstraction of the SCOP and ASTRAL databases. The modules are designed to work as part of the Biopython distribution. Python users can now manipulate and use the SCOP hierarchy from within python programs, and use ASTRAL to return sequences of domains in SCOP, as well as clustered representations of SCOP from ASTRAL.

**Conclusion:**

The modules make the analysis and generation of datasets for use in structural genomics easier and more principled.

## Background

Bioinformatics tools often attempt to automatically predict the unknown properties of a given dataset. Manually curated data is therefore important both for training and for benchmarking new approaches to prediction. One database providing such a manual curation of data is SCOP (the Structural Classification Of Proteins) [1]. SCOP categorises all known protein domains in a hierarchy based upon the domain structure. This hierarchy is principally described by Class, Fold, Superfamily and Family. Crucially, the relationships between proteins grouped at the superfamily level may not be apparent from sequence considerations alone. This makes SCOP a valuable resource when examining the performance of algorithms that detect remote sequence relationships [2].

The ASTRAL [3] Compendium for Sequence and Structure Analysis complements SCOP. ASTRAL provides sequences and structures for each domain in SCOP, and also provides non-redundant subsets of SCOP with preference given to higher quality structures.

The SCOP and ASTRAL databases provide their data in structured files available from the relevant websites. Using this data requires parsing and handling of these files. We present a small and intuitive application programming interface (API) to the SCOP and ASTRAL datasets which allows these databases to be used with a minimum of programming overhead. In the past we have successfully used this API to develop a web-based database of SCOP alignments, S4 [4]. The API described is now distributed as part of the Biopython suite for bioinformatics [5].

## Implementation

The API provides methods that allow the SCOP tree to be queried. Nodes in the SCOP tree can be found using their identification or their position in the tree. In addition, given a particular node, nodes lying on a different level on the tree (ascendents or descendents) can be found.

Each leaf of the tree corresponds to a domain in the SCOP hierarchy. The API uses ASTRAL to provide information on the leaves of the tree, corresponding to domains. For each domain, the API provides its sequence and its membership of non-redundant subsets and sequence as defined by ASTRAL.

## Usage

**Figure 1:**
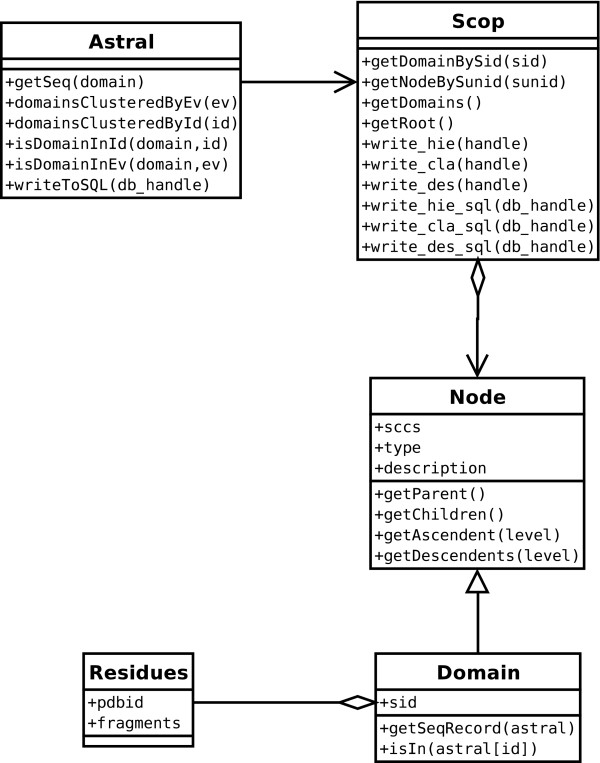
A UML class diagram of classes involved in the interface to SCOP and ASTRAL.

Figure 1 shows a Unified Modelling Language (UML) diagram of the classes and methods involved in the API. Once a Scop object has been instantiated with either a reference to the file or a database, Node objects can be returned by specifying sids (SCOP identifiers that show the pdb identity plus a code that identifies the chain, such as "dlilk_--_") or sunids (SCOP unique identifiers, numerical identifiers assigned to each node in the tree that are guaranteed to be identical across releases, such as "63336"). These Node objects represent all nodes in the tree, including classes, folds, superfamilies and families.

Node objects can be queried for their parent or child objects, and can be queried for relatives further up or down the tree using the getAscendent or getDescendents methods. These methods accept as an argument a string describing the level required, either as the human readable name of the node (e.g. getAscendent ('superfamily')) or using the SCOP conventions for the levels ('cf, 'sf', etc.). Domains are leaves of the SCOP tree and have a special class Domain which stores the sid (e.g. dlh32a2) as well. In addition, each Domain object has a Residues object storing the pdb chain that the domain corresponds to, as well the list of residues from the chain that have been determined to be part of the domain.

The Astral class is an abstraction of the ASTRAL database. ASTRAL provides a FASTA formatted file of all domains in the SCOP database based on PDB records. Using the Biopython framework for handling FASTA files, sequences for SCOP domains can be quickly returned. So, by calling getSeqRecord on a domain with an instance of the Astral class we can retrieve the relevant sequence. ASTRAL also provides FASTA files containing SCOP domains clustered at percent id of residues shared between sequences, or BLAST expect values. The Astral class can parse these files and return Domain objects for each domain in the file. Furthermore, a list of domains for a given percent id (e.g. 10%) or E-value (e.g -10) can be returned using getDomainsClusteredById or getDomainsClusteredByEv.

## Examples

Having downloaded the SCOP parsable files and the ASTRAL scopseq resources, the Astral and Scop objects are instantiated:

>>> from Bio.SCOP import *

>>> scop = Scop(dir_path="...", version=1.67)

>>> astral = Astral (dir_path="...", version=l.67, scop=scop)

Where the ellipses are replaced by suitable paths. We could then find all domains with the same fold as a given domain:

>>> dom = scop.getDomainBySid("dlh32a2")

>>> print dom

dlh32a2 a.3.1.8 (1h32 A:151-261) Di-heme...

>>> fold = dom.getAscendent('fold')

>>> related = fold.getDescendents('domain')

We then use ASTRAL to retrieve a subset of related domains with less than 10% sequence identity.

>>> for r in related:

...   if astral.isDomainInId(r, 10):

...      print astral.getSeq(r)

...

Seq('vdaeavvqqkcischggdltgasapa...

Seq('eadlalgkavfdgncaachagggnnv...

Seq('qadgakiyaqcagchqqngqgipgaf...

A more complex example would be to create a novel dataset to benchmark homology recognition [6]. The authors wished to create a dataset of highly populated sequence diverse superfamilies: those with more than twenty members at less than ten percent sequence identity. Using these modules, such a dataset could be generated in a few lines of code:

>>> superfamilies = scop.getRoot(). getDescendents('superfamily')

>>> dataset = []

>>> for sf in superfamilies:

      desc = sf.getDescendents('px')

      desc = [x for x in desc if astral.isDomainInId(x,10)]

      if len(desc) > 20:

               dataset.append(sf)

## Using MySQL

The database objects provide methods for serialising to a MySQL database handle.

>>> import MySQLdb

>>> db_handle = MySQLdb. connect (...)

>>> scop.write_cla_sql(db_handle)

>>> scop.write_hie_sql(db_handle)

>>> scop.write_des_sql(db_handle)

>>> astral.writeToSQL(db_handle)

This creates the necessary tables and entries; it can then be used to construct Scop and Astral objects.

>>> scop_sql = Scop(db_handle=db_handle)

>>> astral_sql = Astral (db_handle=db_handle, scop=scop_sql)

The advantage of using an SQL approach is that it avoids constructing the entire SCOP tree in memory when the Scop object is created. Instead, database queries are made as and when nodes from SCOP are requested. This avoids the time consuming process of parsing the entire tree, and allows an application using these modules to start quickly.

## Evaluation

The classes have been tested using a unit testing framework, and can correctly parse version 1.61 to 1.67 of the SCOP and ASTRAL databases. Loading and building the SCOP tree from flat files typically takes a few seconds on a modern workstation, although this wait can be avoided by using the MySQL backend.

## Availability

The API is distributed as part of the Biopython suite for bioinformatics .

## Authors' contributions

GEC wrote the original package. JC updated the package with the Astral class and other enhancements with suggestions from GEC and MASS. All authors were involved in the preparation of the manuscript. All authors read and approved the manuscript.
